# Experimental and Analytical Study of Masonry Subjected to Uniaxial Cyclic Compression

**DOI:** 10.3390/ma13204505

**Published:** 2020-10-11

**Authors:** Julian Thamboo, Janaka Bandara, Sithara Perera, Satheeskumar Navaratnam, Keerthan Poologanathan, Marco Corradi

**Affiliations:** 1 Department of Civil Engineering, South Eastern University of Sri Lanka, Oluvil 32360, Sri Lanka; janakachamila44@gmail.com (J.B.); asmperera@gmail.com (S.P.); 2 School of Engineering, Royal Melbourne Institute of Technology, Melbourne 3001, Australia; sathees.nava@rmit.edu.au; 3 Department of Mechanical and Construction Engineering, Northumbria University, Newcastle upon Tyne NE1 8QH, UK; keerthan.poologanathan@northumbria.ac.uk (K.P.); marco.corradi@northumbria.ac.uk (M.C.)

**Keywords:** masonry, compressive strength, cyclic testing, analytical models, lime mortar, plastic strain

## Abstract

Structural evaluation of masonry against dynamic seismic actions invariably requires appropriate cyclic compression constitutive models. However, not many research studies have been dedicated to date to investigate the cyclic compression behaviour of masonry. Therefore, series of experimental investigation followed by analytical model verification were employed in this research to better understand the cyclic compression characteristics of masonry. Twelve masonry wallettes were experimentally tested under cyclic compression loading with different unit-to-mortar assemblies, which are commonly found in masonry structures. The experimental results indicated that the cyclic compression behaviour is greatly influenced by the masonry compressive strength and deformation properties. Thereafter, the ability of five literature analytical models to predict the masonry structural response under cyclic compression loading was investigated. The advantages and limitations of these models are presented and discussed, and the most appropriate analytical model to define the cyclic compression characteristics of masonry has been evaluated and reported. The suggested analytical model is shown to predict the cyclic compression characteristics of different masonry assemblies such as the envelop response, the stiffness degradation, the plastic strain history of the unloading and reloading stages.

## 1. Introduction

Masonry constitutes large percentage of the building stock around the world and it mainly consists of load-bearing walls with the function of resisting gravity actions. Furthermore, within the Reinforced Concrete (RC) framed structures, masonry is used as infill walls and internal partitioning system. These infill walls are considered as non-structural elements, nonetheless they certainly contribute to the lateral load resisting mechanism through diagonal compressive strut-and-tie action to the RC frame [1,2,3,4]. Therefore, understanding the compressive strength characteristics of masonry is an important aspect in the assessment and design of masonry structures. Over the last decades, the compressive strength and the associated deformation characteristics of masonry have been continuously researched for better analyses of the existing structures and develop appropriate design approaches for new construction [5,6,7,8]. 

Primarily the compressive strength and deformation characteristics of various masonry assemblies were assessed through (a) experimental, (b) analytical and (c) numerical methods. Subsequently masonry wallettes/panels and stack bonded prisms have been constructed and tested under monotonic compression to experimentally assess the compression characteristics [9,10,11]. Further, based on the strength and deformation characteristics of constitutive materials (i.e., the units and mortar), analytical solutions have been proposed in the past to determine the compression characteristics of masonry [12,13]. Moreover, with the advent of numerical procedures, there have been few research studies to numerically reproduce the experimental compressive behaviour of masonry [14,15,16,17]. 

Although the monotonic compressive strength characteristics of masonry have been extensively investigated with the aim at developing and/or re-evaluating the strength-based design provisions in standards, the knowledge of the cyclic compressive strength characteristics of masonry is still limited. However, this characteristic is highly important for the seismic or dynamic analysis of masonry structures. The research studies dedicated to experimentally or analytically examine the masonry cyclic compression characteristics are rarely compared to the studies dedicated to investigate the monotonic compression characteristics. Only few studies exist across different masonry types [18,19,20,21,22,23,24,25]. It is commonly understood that the masonry cyclic compressive strength is moderately less than the corresponding monotonic strength, and the axial deformation capacity derived from cyclic tests is relatively higher than the equivalent monotonically tested masonry. 

To study the masonry cyclic behaviour, experimental campaigns are not always viable to conduct. Therefore, analytical solutions are needed for a better evaluation of the cyclic characteristics of different masonry assemblies. However, a limited number of studies exist on the analytical modelling of masonry under cyclic loading. Using their own test results for calibration, Subramaniam and Sinha [25] proposed empirical formulations to simulate the plastic response, the unloading and reloading cyclic behaviour of brick masonry. Crisafulli [26] proposed a cyclic analytical model for masonry to simulate the infill masonry walls based on the cyclic analytical models proposed for concrete. A study by Sima et al. [27] outlined a comprehensive analytical model and calibrated it against the experimental data from Naraine and Sinha [18]. Recently, Facconi et al. [28] improved the Crisafulli [26] model constants with the smeared rotating crack approach. Most of those cyclic analytical models were derived from related cyclic analytical models of concrete [29,30,31,32,33,34]. For example, Mendola and Papia [35] developed a generalised cyclic analytical model for masonry from the concrete constitutive model proposed by Mander et al. [29]. Subsequently, the approaches and formulations proposed to characterise the masonry cyclic characteristics overlap and differ with each other in terms of the treatment of envelop curves, plastic strain, unloading and reloading history. These aspects are explained more in detail in Section 2 and Section 4. 

In summary, the available research studies on the characterisation of the cyclic constitutive behaviour of masonry under compression are limited. Also, among the few constitutive models proposed to define the cyclic compression behaviour of masonry, the approaches and formulations proposed to predict each component of the cyclic compression characteristics differ from each other. Therefore, the aim of this research was to evaluate the most appropriate analytical model for masonry subjected to cyclic compression. In this research five literature cyclic analytical models were selected and their ability to characterise each aspect of the cyclic compression stress–strain curves was studied. Thereafter, an experimental cyclic compression testing programme on masonry wallettes was conducted to generate diverse experimental data and to verify the applicability of the selected analytical models. Finally, the most appropriate analytical model has been identified based on its ability to reproduce the experimental behaviour on different aspects of the cyclic stress–strain characteristics. 

## 2. Cyclic Constitutive Models

**Figure 1:**
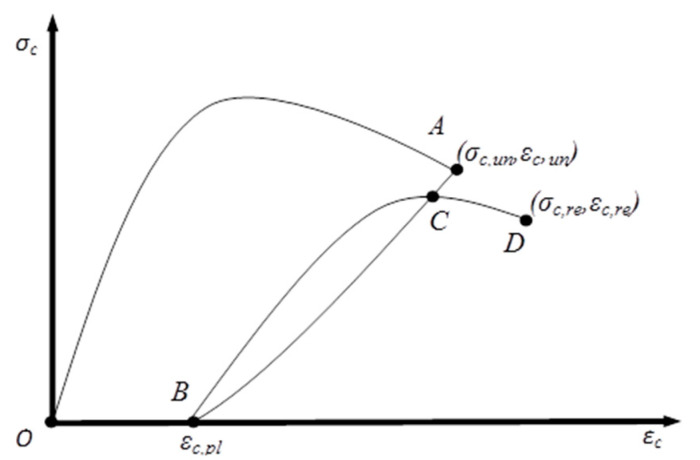
Typical stress–strain curve of masonry under cyclic compression.

The general cyclic response of quasi-brittle construction materials such as masonry and concrete under uniaxial compression loading, unloading and reloading is well understood by the research community. The typical cyclic stress–strain curve of masonry with the assumption of zero tensile strength is shown in Figure 1. Point O (*0*, *0*) denotes the origin of the curve and Point A (*σ_c,un_, ε_c,un_*) corresponds to the bringing of unloading curve. The line AB is the unloading curve that ends at point B (*0*, *ε_c,pl_*). It has to be mentioned that for relatively moderate unloading stresses, point B meets the abscissa slightly offset to the origin, which is refereed as “plastic strain” or “residual strain” (*ε_c,p_*_l_). This is one of the essential parameters in the cyclic stress–strain behaviour of masonry under cyclic compression. The reloading curve begins with slightly lower stiffness and meets the previous unloading curve at C, which is generally referred as “Common Point”. Afterwards the reloading branch ends at point D (*σ_c,re_, ε_c,re_*) and the same set of behaviour continues until failure of the masonry.

Even though, the overall characteristics of the cyclic response are well recognised: The treatment of the individual aspects of the stress–strain response, such as the envelop curve, plastic strain, and unloading and reloading curves, differ among the analytical models reported in the existing literature. Therefore, in this research, five commonly referred analytical models were initially considered and reviewed, as these have been consistently discussed and used in the seismic and dynamic analyses of masonry structures in the past. These five analytical models are briefly summarised in the following sub-sections, highlighting the commonalities and differences in the formulations given to model each aspect of the cyclic stress–strain curve. The formulations given in those models are outlined in Table 1, Table 2, Table 3, Table 4 and Table 5. 

### 2.1. Subramaniam and Sinha Model

The analytical stress–strain model proposed in this research study is empirical in nature. The formulations in the model were developed from their own testing programme [12]. Masonry wallettes were made with solid clay bricks and cement-sand mortar. The mean compressive strengths of brick and mortar were 13.1 MPa and 6.1 MPa, respectively. Primarily, a curve fitting method was followed to develop the envelop, unloading and reloading curves and to estimate the post-elastic behaviour. Consequently, third and fourth order polynomial functions were proposed to fit the unloading and reloading curves of the cyclic model. Table 1 presents the empirical formulations proposed to define each characteristic in model. It can be noted that different empirical constants are given in each part of the cyclic stress–strain. However, it could be concluded that the analytical model was only validated against one set of experimental data; therefore the applicability of the model with different types of masonry assemblies needs to be better verified. 

### 2.2. Crisafulli Model 

A more refined analytical model for cyclic masonry behaviour was proposed by Crisafulli [26] compared to model proposed by Subramaniam and Sinha [25]. The envelop curve is defined with a quadratic formulation, where five different parameters are required to characterise the envelop curve. The envelop curve is defined using two formulations to represent the pre-peak and post-peak portions. A common nonlinear continuous formulation is recommended for the unloading and reloading curves. Nine empirical constants are required for the unloading and reloading formulation. Therefore, fourteen parameters are in total required to complete the cyclic stress–strain curve as presented in Table 2. This model is able to simulate the masonry unloading and reloading cyclic behaviour. The model has been validated against the experimental data of Naranie and Sinha [18], and the partial unloading and reloading cyclic behaviour was also confirmed using test results from concrete cylindrical samples.

### 2.3. Mendola and Papia Model 

A generalised stress–strain constitutive model for concrete and masonry is proposed in this study. Table 3 outlines the treatment of each characteristic of the proposed model. In the model it is assumed that the cyclic envelop curve follows the same trend of a monotonic compression test. This assumption can be slightly un-conservative, as recent studies indicate that the cyclic compressive strength is smaller than the monotonic strength. Simplified formulation to predict the unloading and reloading branches have been proposed. The determination of the residual strain was proposed from the unloading strain and stiffness levels. The model requires thirteen parameters to be calibrated. This model was also validated against the experimental cyclic compression test data of Naranie and Sinha [18].

### 2.4. Sima et al. Model 

The formulation to simulate the masonry cyclic behaviour was derived from the concrete cyclic analytical model proposed by the same researchers [24]. The envelop curve of the model is represented by the compression damage using a parameter which characterises the material damage in each cycle. The proposed exponential formulations represent the unloading and reloading curves as given in Table 4. The model requires determining several damage parameters to construct the cyclic constitutive behaviour of masonry. The reloading curve is characterised by a line in this model, which is quite different to what is commonly observed in experimental studies. Further empirical way of determining the plastic strain from the level of unloading strain was used in the model. Finally, this analytical model was also validated against the experimental data of Naraine and Sinha [18]. 

### 2.5. Facconi et al. Model

The proposed analytical model was aimed at stimulating the smeared rotating crack of masonry structures under cyclic loading. The envelop curve is defined using two formulations to represent pre-peak and post-peak behaviour. The formulation used to define the unloading and reloading curve was mainly taken from Crisafulli [26]. The idea of reloading curve and stiffness for the model was taken from concrete models [28,30]. Also, an empirical formulation similar to that given in Subramaniam and Sinha [25] was used to predict the plastic strain as the function of unloading strain. The proposed model needs thirteen numeric parameters to completely define the constitutive model as given in Table 5. The model was validated against the experimental data developed in the authors’ research programme. The masonry wallettes were made of clay brick and cement–lime–sand mortar and subjected to uniaxial cyclic loading. Further the test results of Naraine and Sinha [18], Galman and Kubica [20], and Oliveria et al. [22] were used to verify the applicability of the proposed model.

## 3. Experimental Programme

The experimental studies on the cyclic compression behaviour of masonry are limited in terms of the types of masonry assemblies used; they are mostly made of relatively high compressive strength clay bricks and mortar. However, there are diverse types of masonry assemblies in structures with different strength and deformation characteristics of its constituents (i.e., units and mortar). It can be generally categorised that the solid brick masonry is made primarily with four different combinations of units and mortar. They are (1) comparatively higher strength units and lower strength mortar, (2) comparatively higher strength mortar and lower strength units, (3) relatively low strength units and mortar and (4) comparatively higher strength units to relatively higher strength mortars. Subsequently, the mechanical characteristics of masonry made with diverse unit-to mortar combinations are different to each other, as their individual characteristics contribute to the overall compression behaviour of the masonry [36,37,38]. Thus, an appropriate analytical model should be able to predict the cyclic compression behaviour of different masonry assemblies. Subsequently, to assess the cyclic compression response of the different masonry assemblies, an experimental programme has been planned to generate cyclic compression test data of different combinations of masonry assemblies.

This experimental programme was part of an ongoing research study at South Eastern University of Sri Lanka, aimed at investigating the monotonic and cyclic compression behaviour of different masonry assemblies. Twelve masonry wallettes were constructed and tested under cyclic compression to develop experimental data. Only the brief details on the used constitutive materials, construction procedure of the wallettes and the cyclic testing protocol are given in this paper; further information can be referred in Thamboo and Dhanasekar [39,40,41]. 

### 3.1. Materials

Mainly two types of clay bricks were selected to construct the masonry wallettes. Those clay bricks have been chosen from the local construction industry. They are denoted as CB1 and CB2. The length× width× height of CB1 and CB2 bricks are 200 mm × 95 mm × 65 mm and 210 mm ×100 mm × 60 mm respectively. The mean compressive strengths of CB1 and CB2 bricks are determined as according to BS EN 772-1 [42], using a displacement-controlled testing mode to determine the elastic moduli. Six samples were tested in each brick type to determine the mean compressive strengths and elastic moduli. The obtained mean compressive strength of bricks was 5.1 MPa (Coefficient of Variation (COV) = 9.5%) and 15.5 MPa (COV = 5.8%), for CB1 and CB2 type, respectively. These two types of bricks were purposely selected to represent relatively low (CB1) to high (CB2) unit strength combinations in the experimental programme. Further mean elastic modulus (Young’s modulus) of the CB1 and CB2 bricks were 4123 MPa (COV = 3.6%) and 9755 MPa (COV = 3.6%) correspondingly.

Further two types of mortars were used to construct the masonry wallettes. Ordinary Portland cement (CM) and natural hydraulic lime (NHL) were used as binders to prepare the two types of mortars. Both mortar mixes were prepared at a binder-to-filler ratio of 1:3 by volume. While constructing the masonry wallettes, mortar 100 mm diameter cylinders (height = 200 mm), according to ASTM C780-18a [43], were casted and tested under compression to determine the mortar compressive strength. An extensometer was also attached to the cylinders to capture the axial deformation and to determine the elastic modulus of mortar. Six mortar cylinders were casted for each mix to determine the mean compressive strengths and elastic moduli. The elastic stress was considered as one-third of the peak stress of the stress–strain curve of the mortar, and the elastic strain was taken as the matching strain obtained at one-third of the peak stress. The mean compressive strength of the NHL and CM mortars were 2.0 MPa (COV = 8.9%) and 13.9 MPa (COV= 6.5%), respectively. Furthermore, the mean elastic modulus of the NHL and CM mortars were 1402 MPa (COV = 3.6%) and 9564 MPa (COV = 5.6%) respectively. Hence it can be concluded that these two mortar types represent a relatively low (NHL) and high (CM) strength mortars. 

### 3.2. Wallette Construction

In total, four units-to-mortar masonry wallette combinations were used for cyclic compression tests. Subsequently, twelve masonry wallettes were constructed with three specimens per each units-to-mortar combination. As explained before, the unit-to-mortar combinations were deliberately planned to represent different masonry assemblies of low mortar strength (i.e., ~5 MPa) to low unit strength (~5 MPa), low mortar strength to higher unit strength (i.e., ~15 MPa), higher mortar strength (i.e., ~15 MPa) to lower unit strength and higher mortar strength to higher unit strength. The wallettes were built as per BS EN 1052-1 [44] provisions. 

Double-wythe brickwork wallettes (with a thickness of 200 mm and 230 mm for CB1 and CB2 series wallettes respectively) were used for testing. The dimensions of the wallettes combinations slightly varied from each other as the type of brick used was varied. The constructed wallettes dimensions are presented in Table 6. An experienced mason was assigned to construct the wallettes. The thickness of the mortar bed and head joint was about 10 mm. The testing arrangement is presented in Table 6. The constructed wallette specimens were air cured at the laboratory prior to the testing. Subsequently, testing was carried out after 28 days of constructing the wallettes. An alpha-numeric index was used to denote each test: CB1 and CB2 are used to identify the brick type; a second set of letters denotes the used mortar type (NHL and CM). 

### 3.3. Instrumentation and Testing

**Figure 2:**
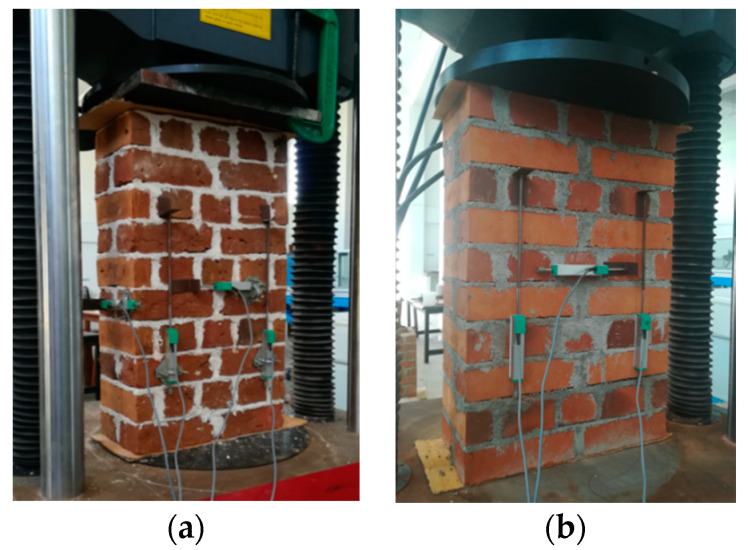
(**a**) Testing of natural hydraulic lime (NHL) mortared wallettes and (**b**) Testing of ordinary Portland cement (CM) mortared wallettes.

The cyclic compression testing was carried out using a 1000 kN capacity servo-controlled universal testing machine (UTM, WAW1000E, Jinan, China). The testing set up of the NHL and CM mortared wallettes is shown in Figure 2a,b, respectively. For each wallette testing, four displacement transducers were vertically fixed on both faces (two per face) to record the axial deformation. Further two displacement transducers were horizontally fixed on the face of the wallette (one per face) to measure the lateral deformation.

The cyclic loading protocol was assigned from the load-displacement responses of the monotonic and cyclic testing previously carried out by the first author and reported in Thamboo and Dhanasekar [40]. From the monotonic load-displacement testing curves, the elastic, hardening, peak and ultimate points were recognised and the cyclic protocol was assigned for each combination of wallette testing. The rate of loading was given as 0.25 mm/min for all the reloading and unloading stages in the cyclic protocol. The loads and displacements were recorded using a data acquisition system.

### 3.4. Experimental Results

The experimental results are presented and discussed in terms of failure modes, compressive strengths and cyclic stress–strain curves acquired for each unit-to-mortar combination in the succeeding sub-sections.

#### 3.4.1. Failure Modes

**Figure 3:**
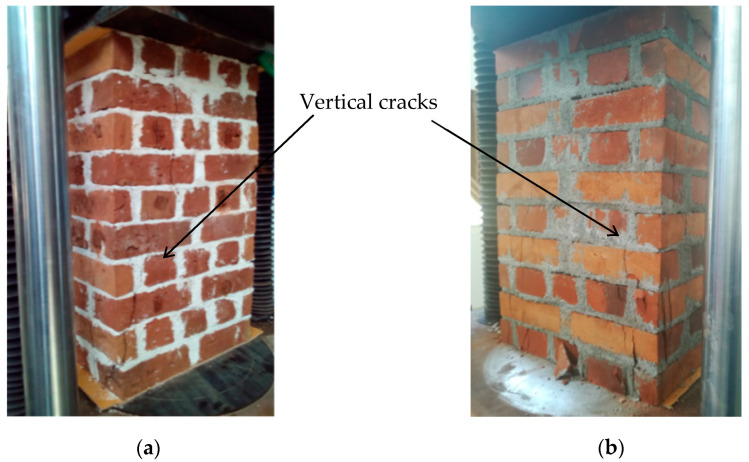
Failure modes of wallettes under cyclic compression (**a**) CB1-NHL mortared wallettes and (**b**) CB2-CM mortared wallettes.

The failure modes of the wallettes under cyclic compression loading are shown in Figure 3. The failure modes were generally distinguished with vertical cracks started at brick to mortar interface (head joints) [20]. This phenomenon is the consequence of incompatible elastic properties of the brick and mortar, and it typically induces vertical parallel cracks in the bricks under axial compressive stress. Furthermore, it has to be mentioned that the mortar strength of the CB1-CM series is relatively higher than the brick strength. Therefore, the unit could have dilated more than the mortar, nevertheless the ultimate failure pattern of the CB1-CM wallettes are similar to other wallette combinations tested. Hence variation in unit and mortar types did not change the failure pattern of the wallettes under compression [45,46]. Commonly the initial cracks were started to appear at about 50% to 70% of the peak load, thereafter cracks got wider and propagated with nonlinear load resistance behaviour until ultimate failure. The testing was stopped for the safety of the instrumentation after the wallettes exhibited quite significant cracking. 

#### 3.4.2. Strength and Deformation

The mean cyclic compressive strengths are given in Table 7 and the corresponding COVs are given in parentheses. It can be noted that the CB2 series wallettes have shown a higher cyclic compressive strength than the CB1 series wallettes as their unit strengths are higher than the CB2 series wallettes. Furthermore, considering the mortar type, the CM mortared wallettes have shown slightly higher compressive strength than the corresponding NHL mortared wallettes. However, the strength increment (22–24%) is not substantial when compared to the strength difference between the mortars (695%). This effect is quite well known: Past studies have demonstrated that the compressive strength of mortar does not significantly contribute to the compressive strength of the masonry itself, as the mortar is under a confined state [47,48,49].

**Figure 4:**
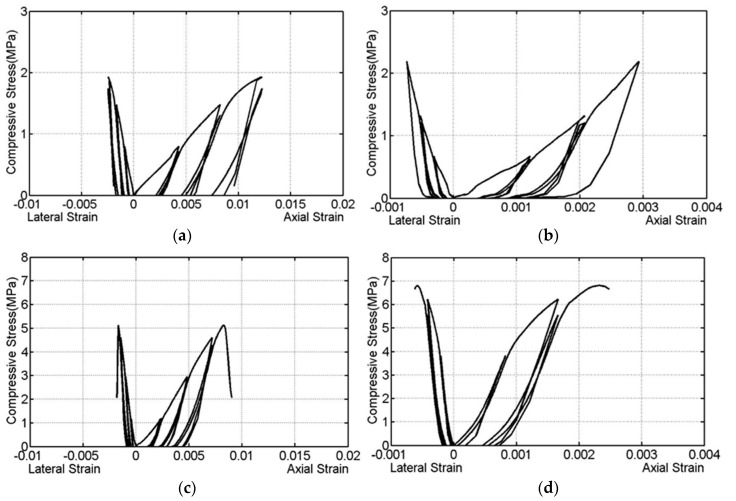
Cyclic compressive stress–strain curves of the wallettes under compression (**a**) CB1-NHL, (**b**) CB1-CM, (**c**) CB2-NHL, and (**d**) CB2-CM.

Figure 4 shows the stress–strain curves obtained in the cyclic testing of wallettes. The typical (i.e., closed to average strength) cyclic stress–strain curve for each combination is presented in Figure 4 as showing all curves would be difficult for any comprehension. It can be observed that the non-linear characteristics of the masonry wallettes initiated roughly around 40% to 50% of the peak compressive stress. This was also connected with the beginning of minor vertical cracks in the wallettes. The buildup of non-reversible axial strains in the wallettes; especially after nearly 40% peak stress can also be noted. Also the strength and stiffness degradation at each step and cycle can be noticed. It designates that a progressive damage has happened in each loading cycle in the wallettes. 

Further, the CB1-series wallettes have exhibited comparatively more axial deformability than the CB2-series wallettes as the elastic modulus of CB1 bricks was comparatively smaller than the CB2 bricks. Also, it can be concluded that the mortar deformation characteristics influenced significantly the axial cyclic deformation characteristics of masonry as the NHL mortared wallettes have shown higher strain than CB mortared wallettes. The mortar strength does not significantly contribute to the masonry compressive strength; however, its deformation characteristics certainly contribute to the axial masonry deformation under compression.

Moreover, the deformation properties of the masonry wallettes, such as (1) Young’s modulus, (2) Poisson’s ratio, (3) elastic strain, (4) peak strain, and (5) ultimate strain were calculated from the stress–strain curves. These are presented in Table 7 with the corresponding COVs. The Young’s modulus of the masonry wallette was computed at the one-third of the peak stress and matching strain. The Poisson’s ratios were calculated from the elastic axial strain values and the relevant lateral strain values. The peak strain was determined in correspondence of the peak compressive stress. The ultimate strain was taken equivalent to the 80% of the post-peak stress. 

The elastic moduli of the CB1 and CB2 series wallettes vary between 132 MPa and 1398 MPa and between 446 MPa and 3342 MPa respectively. Further the Poisson’s ratio ranges between 0.15 to 0.18. Moreover, the elastic, peak and ultimate strains measured among the tested wallettes indicate clear differences between the deformation characteristics of CB1 and CB2 series wallettes and also between NHL and CB mortared wallettes. 

## 4. Verification of Analytical Models with Experimental Data

As presented in Section 2, different analytical models were proposed in the past to predict the cyclic compression characteristics of masonry. However, most of these models have been independently developed and verified against limited experimental results. The applicability of these analytical models to predict different masonry assemblies is not well examined. Hence, in order to assess the predictability of these analytical models and to evaluate the most appropriate one, the experimental cyclic compression data generated in this research were used. Additionally, experimental literature studies were also employed to diversify and increase the experimental base for comparison purposes.

Subsequently, the experimental cyclic compression stress–strain curves obtained for the four masonry unit-to-mortar combinations in this research (CB1-NHL, CB1-CM, CB2-NHL, and CB2-CM) and two cyclic stress–strain literature curves were considered. The two cyclic stress–strain literature curves were taken from the experimental studies of Naraine and Sinha [18] and Facconi et al. [28]. The characteristics of the cyclic compression stress–strain curves comprised of envelop curve, plastic strain, and unloading/reloading curves. Therefore, the ability to predict these individual aspects in the proposed analytical cyclic stress–strain models were verified against the generated experimental data and explained in the following sub-sections.

### 4.1. Envelop Curve

**Figure 5:**
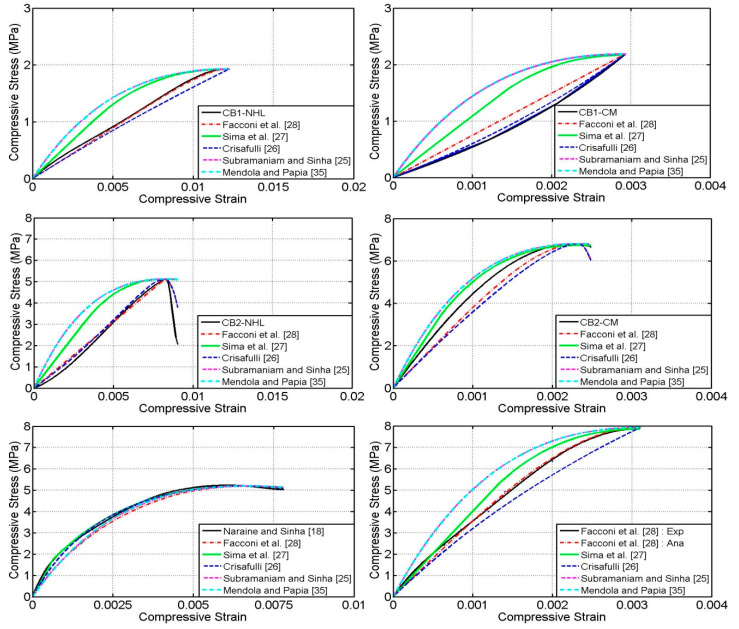
Comparison of experimental and analytical envelop curves.

Figure 5 shows the comparison of experimental and analytical envelop curves. In general, all the analytical envelop curves follow a similar trend as experiment curves; however their predictability of envelop stress/strains values and the stiffness were different to each other. It can be noted that the analytical models proposed by Subramaniam and Sinha [25], Sima et al. [27] and Mandola and Papia [35] over predict the stiffness of the envelop curve. 

Nevertheless, the analytical models proposed by Crisafulli [26] and Facconi et al. [28] reasonably predict the experimental envelop curves, especially the model suggested by Facconi et al. [28] accurately predicted all the experimental envelop curves. The reason for this quite accurate prediction is due their assimilation of two set envelop curve formulations (pre-peak and post-peak), whereas the other three models outlined single formulation. Especially envelop curve model proposed by Facconi et al. [28] was taken from Hoshikuma et al. [50] and recently Thamboo and Dhanasekar [40] have shown that this envelop model predict the behaviour of relatively low strength masonry as well. Thus it can be stated that this model is capable of predicting the envelop curves of different masonry assemblies verified in this research.

### 4.2. Plastic Strain

**Figure 6:**
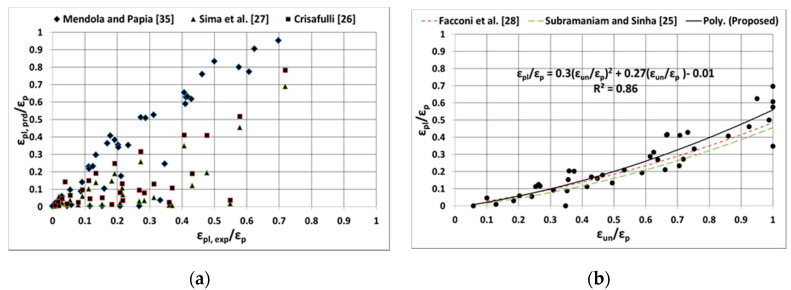
Comparison of experimental and analytical plastic strains (**a**) Mendola and Papia [35], Sema et al. [27] and Crisafulli [26] and (**b**) Facconi et al. [28] and Subramaniam and Sinha [25].

Furthermore, predicting the plastic strain is an important step in defining the shape of the unloading curve and it also indicates the level of accumulated damage in the masonry during the cyclic loading. Previous experimental and analytical studies on masonry and concrete indicate that the plastic strain is depended on the level of strain at which the unloading starts; therefore, all the proposed analytical model formulations used to predict the plastic strain are primarily based on the unloading strain and the associated parameters. However, the way the formulations are treated to predict the plastic strain is different to each other. The plastic strain formulation proposed by Crisafullu [26], Sima et al. [27] and Mendola and Papia [35] are relatively complex, as they not only require to determine the unloading strain, but are also dependent on several parameters (e.g. initial secant modulus, unloading stress, unloading modulus, and empirically derived coefficients). However, the plastic strain formulation suggested by Subramaniam and Sinha [25] and Facconi et al. [28] are empirical in nature; they are only dependent on the unloading strain. Nonetheless, the plastic strains predicted using the analytical models and their corresponding experimental data are compared and presented in Figure 6. For uniform comparison purposes, the experimental and analytical plastic strain data were normalised by means of dividing them by the peak strain.

It can be noted from Figure 6a, that the predictability of plastic strain by the analytical formulations proposed by Crisafulli [26], Sima et al. [27] and Mendola and Papia [35], varies considerably with the experimental data. Especially the formulation given in Mendola and Papia [25] over predicts and the formulation proposed by Crisafulli [26] relatively under predicts the plastic strain. However, it can be also observed from Figure 6b, that the empirical formulations proposed by Subramaniam and Sinha [25] and Facconi et al. [28] match relatively well with the experimental data. Subsequently, a regression analysis was carried out using the experimental data to predict the plastic strain using unloading strain, and the regression formulation obtained matches well with the formulations proposed by Subramaniam and Sinha [25] and Facconi et al. [28]. Therefore, it can be mentioned that the formulations proposed by Subramaniam and Sinha [25] and Facconi et al. [28] can predict the plastic strain quite accurately for the masonry types considered in this study and they are simple to use as well. Therefore, they can be effectively used in the development of cyclic stress–strain of different masonry assemblies.

### 4.3. Unloading and Reloading Curves

The characteristics of the unloading and reloading curves of the cyclic stress–strain response depend of various parameters and they are quite complex to accurately predict. Normally, the unloading curves start from the envelop curve and end at plastic strain, in the case of partial unloading; it may be at any point in between abscissa and unloading point. Therefore appropriate predictions of envelop and plastic strain characteristics are important in defining the unloading and reloading curves. Subsequently it can be noted from the Section 4.1 and Section 4.2, the analytical formulation proposed by Facconi et al. [28] predicts the envelop and plastic strain relatively well, whereas other models deviate to predict one or other aspects. It has to be mentioned that the unloading and reloading formulations used by Facconi et al. [28] are almost the same as the formulations given in Crisafulli [26]. 

**Figure 7:**
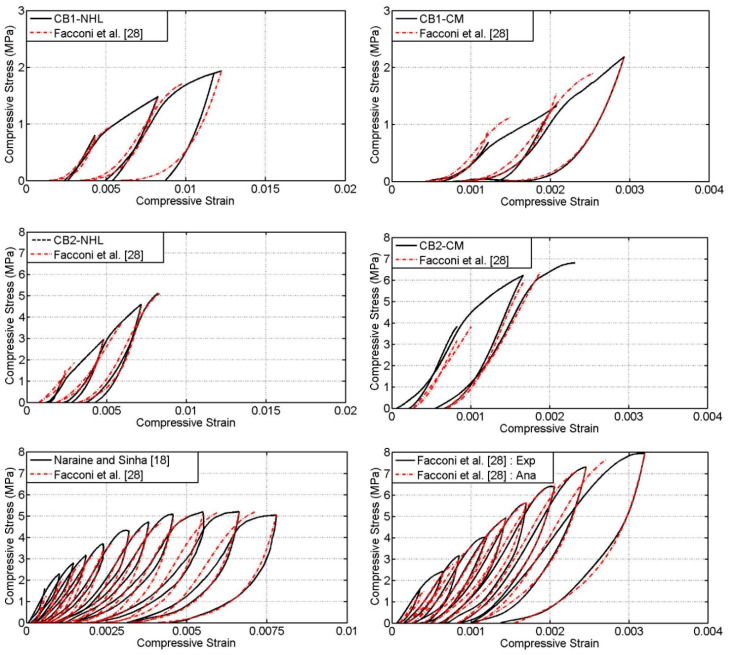
Comparisons of experimental and analytical unloading and reloading curves.

Subsequently the formulations given in Facconi et al. [28] have been used to analytically predict the unloading and reloading curves and compared with the experimental curves. Figure 7 shows the comparison between the analytical and experimental unloading and reloading curves. It can be noted that the model predicts the unloading and reloading characteristics relatively well despite of the differences in types of masonry assemblies used. Therefore, it can be stated that the analytical formulations proposed by Facconi et al. [28] or Crisafulli [26] can be effectively used to predict the unloading and reloading portions of the stress–strain response of masonry under cyclic compression. 

### 4.4. Overall Comparisons

**Figure 8:**
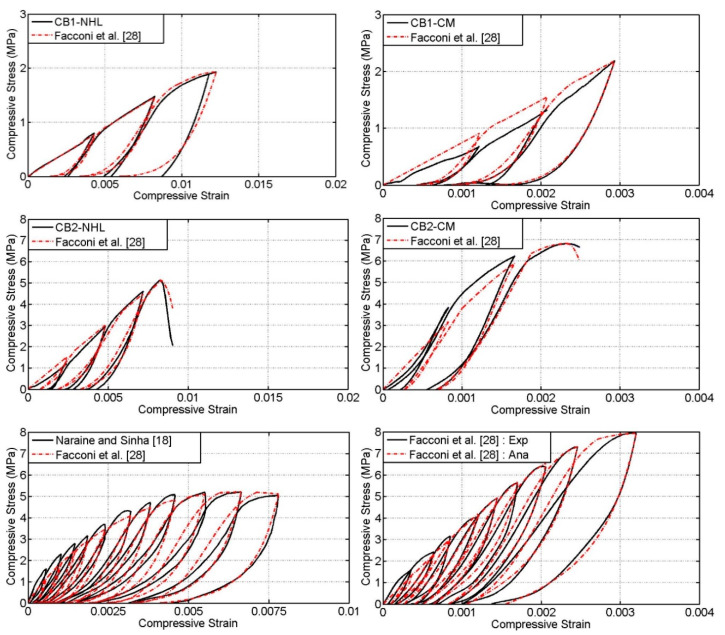
Comparison of experimental cyclic stress–strain curves with analytical curves of Facconi et al. [28].

Since the model proposed by Facconi et al. [28] has show to predict envelop curve, plastic strain and unloading and reloading portions comparatively well, therefore the complete cyclic stress–strain predictions of this model has been compared with the experimental cyclic stress–strain responses and presented in Figure 8. It can be noted that the model follows relatively well the experimental cyclic responses of different unit-to-mortar masonry assemblies taken for comparison. It has to be highlighted that the model is also capable to predict the partial unloading and reloading stages. Thus, it can be concluded that it can be used to estimate the cyclic compression response of different masonry assemblies.

## 5. Summary and Conclusions

This research was aimed to determine the most appropriate model to define the cyclic compression characteristics of masonry using experimental and analytical verifications. Subsequently the systematic steps taken to achieve the aim of the research study were: (1) Reviewing the available analytical models that proposed to define the cyclic compression characteristics of masonry, (2) experimental cyclic testing of four different masonry assemblies under cyclic compression to generate diverse data and (3) verifying the analytical models against the experimental data generated to determine the most appropriate model considering all the aspects of the cyclic stress–strain response of masonry. The following conclusions have been drawn from the experimental data gathered and the analytical verification made in this research:

(1) The review of the available analytical models to define the cyclic compression stress–strain characteristics of masonry discloses that they are developed based on the concrete cyclic analytical models. These models differently treat the components of the cyclic stress–strain response such as the envelop curve, the plastic strain, the unloading and reloading phases;
(2) The cyclic testing results of four possible units-to-mortar combinations reveal that the cyclic compression stress–strain characteristic of masonry is greatly influenced by the characteristics of the constitutive materials;
(3) The cyclic stress–strain predications of the five different analytical models tend to differ from each other. For the envelop curve, the analytical formulations proposed by Crisafulli [26] and Facconi et al. [28] match relatively well with the experimental curves considered. The plastic strain values are well predicted by the analytical formulations given in Subramaniam and Sinha [25] and Facconi et al. [28]. Subsequently the unloading and reloading curves using the formulations given in Facconi et al. [28] show a good agreement with the experimental curves;
(4) In general, the cyclic stress–strain analytical model given in Facconi et al. [28] tends to match well with the experimental data considered in this research in all aspects. Whereas other models have shown to match the experimental data in few selected aspects as most of them were developed and verified against limited test data. Hence Facconi’s model can be used consciously to simulate the cyclic compression stress–strain response of different masonry assemblies.

## Figures and Tables

**Table 1:** Constitutive model for masonry under cyclic compression: Subramaniam and Sinha [25].

**Envelop Curve**	σc=εcβe(1−εcα)
**Unloading and Reloading Curves**	$\sigma_{c}=0.84\left(\frac{\varepsilon }{\varepsilon_{cp}}-\frac{\varepsilon_{c,pl}}{\varepsilon_{cp}}\right)-0.29{\left(\frac{\varepsilon }{\varepsilon_{cp}}-\frac{\varepsilon_{c,pl}}{\varepsilon_{cp}}\right)}^{2}-1.72{\left(\frac{\varepsilon }{\varepsilon_{cp}}-\frac{\varepsilon_{c,pl}}{\varepsilon_{cp}}\right)}^{3}-1.27{\left(\frac{\varepsilon }{\varepsilon_{cp}}-\frac{\varepsilon_{c,pl}}{\varepsilon_{cp}}\right)}^{4}$ $\sigma_{c}=0.38\left(\frac{\varepsilon }{\varepsilon_{cp}}-\frac{\varepsilon_{pl}}{\varepsilon_{cp}}\right)+0.61{\left(\frac{\varepsilon }{\varepsilon_{cp}}-\frac{\varepsilon_{pl}}{\varepsilon_{cp}}\right)}^{2}$
**Plastic Strain**	$\varepsilon_{c,pl}=0.007+0.208\varepsilon_{c}+0.256\varepsilon_{c}^{2}$

**Table 2:** Constitutive model for masonry under cyclic compression: Crisafulli [26].

**Envelop Curve**	$\sigma_{c}=\sigma_{cp}\frac{A_{1}\frac{\varepsilon_{c}}{\varepsilon_{cp}}+\left(A_{2}-1\right){\left(\frac{\varepsilon_{c}}{\varepsilon_{cp}}\right)}^{2}}{1+\left(A_{1}-2\right)\frac{\varepsilon }{\varepsilon_{p}}+A_{2}{\left(\frac{\varepsilon_{c}}{\varepsilon_{cp}}\right)}^{2}}$; $\varepsilon_{c}\leq \varepsilon_{cp}$ $\sigma_{c}=\sigma_{\mathrm{cp}}\left[1-{\left(\frac{\varepsilon_{c}-\varepsilon_{cp}}{\;\varepsilon_{cu}-\;\varepsilon_{cp}}\right)}^{2}\right];\varepsilon_{c}\geq \varepsilon_{cp}$ $A_{1}=\frac{E_{0}\times \varepsilon_{cp}}{\sigma_{cp}}$; $A_{2}=1-\;A_{1}\frac{\varepsilon_{cp}}{\varepsilon_{cu}}$
**Unloading and Reloading Curves**	$\sigma_{c}=\sigma_{c1}+\left(\sigma_{c2}-\sigma_{c1}\right)\frac{B_{1}\chi +\chi^{2}}{1+B_{2}\chi +B_{3}\chi^{3}}$ $\chi =\frac{\varepsilon_{c}-\varepsilon_{c1}}{\varepsilon_{c2}-\varepsilon_{c1}}$; $E_{s}=\frac{\sigma_{c2}-\sigma_{c1}}{\varepsilon_{c2}-\varepsilon_{c1}}$; $\;B_{1}=\frac{E_{1}}{E_{s}}$; $\;B_{2}=B_{1}-B_{3}$ $B_{3}=2-\frac{E_{0\times }\left(1+B_{1}\right)}{E_{s}}$
**Plastic Strain**	$\varepsilon_{c,pl}=\varepsilon_{c,un}-\frac{\left(\varepsilon_{c,un}-\frac{\beta_{a}\sigma_{cp}}{E_{0}}\right)}{\sigma_{un}-\beta_{a}\sigma_{cp}}$

**Table 3:** Constitutive model for masonry under cyclic compression: Mendola and Papia [35].

**Envelop Curve**	$\sigma_{c}=\frac{A\varepsilon_{c}+\left(D-1\right)\varepsilon_{c}{}^{2}}{1+\left(A-2\right)\varepsilon_{c}+D\varepsilon_{c}{}^{2}}$
**Unloading and Reloading Curves**	$\sigma_{c}=\varepsilon_{c}k_{j}^{\left(1-\varepsilon_{c}{}^{-m_{j}}\right)}$
**Plastic Strain**	$\varepsilon_{c,pl}=\varepsilon_{c,un}-\frac{\sigma_{c,un}}{E_{s,un}}$

**Table 4:** Constitutive model for masonry under cyclic compression: Sima et al. [27].

**Envelop Curve**	$\sigma_{c}=\varepsilon_{c}E_{0}$ $\sigma_{c}=\left(1-\delta_{c}^{-}\right)\varepsilon_{c}E_{0}$
**Unloading and Reloading Curves**	$\sigma_{c}=D_{1}e^{D_{2}\left(1-\frac{\varepsilon_{c}-\varepsilon_{c,pl}}{\varepsilon_{pc-}\varepsilon_{c,pl}}\right)}E_{0}\left(\varepsilon_{c}-\varepsilon_{c,pl}\right)$; Unloading $\sigma_{c}=\varepsilon \times E_{s,re}$; $E_{s,re}$= −0.698 × $\delta_{c,un}$ + 0.934; Reloading
**Plastic Strain**	$\varepsilon_{c,pl}=-36.9\delta_{c,un}^{3}+82.2\delta_{c,un}^{2}-66.4\delta_{c,un}+21.4$

**Table 5:** Constitutive model for masonry under cyclic compression: Facconi et al. [28].

**Envelop Curve**	$\sigma_{c}=\varepsilon_{cp}\left[1-\frac{1}{n}\varepsilon_{c}{}^{n-1}\right]$ $\sigma_{c}=\sigma_{\mathrm{cp}}\left[1-{\left(\frac{1-\varepsilon_{c}}{0.5}\right)}^{2}\right]$
**Unloading and Reloading Curves**	$\sigma_{c}=\sigma_{c1}+\left(\sigma_{c2}-\sigma_{c1}\right)\frac{B_{1}\chi +\chi^{2}}{1+B_{2}\chi +B_{3}\chi^{3}}$
**Plastic Strain**	$\varepsilon_{c,pl}=0.235{\left(\varepsilon_{c,un}\right)}^{2}+0.25\left(\varepsilon_{c,un}\right)$

**Table 6:** Test matrix and dimensions of the specimens.

Unit Type	Mortar Type	Specimen Notation	Wallette Dimensions Length × Width × Height (mm)	Number of Tested Wallettes
CB1	NHL	CB1-NHL	410 × 95 × 740	3
CB1	CM	CB1-CM	410 × 95 × 740	3
CB2	NHL	CB2-NHL	410 × 200 × 740	3
CB2	CM	CB2-CM	410 × 200 × 740	3

**Table 7:** Cyclic compressive strength and deformation properties.

Specimen Notation	Compressive Strength (MPa)	Young’s Modulus (MPa)	Poisson’s Ratio (-)	Elastic Strain (mm/mm)	Peak Strain (mm/mm)	Ultimate Strain (mm/mm)
CB1-NHL	1.93 (12.7)	132 (13.7)	0.15 (11.7)	0.006 (10.1)	0.015 (17.6)	0.018 (10.3)
CB1-CM	2.36 (12.3)	934 (8.5)	0.17 (16.6)	0.0012 (18.5)	0.0032 (11.0)	0.0038 (11.3)
CB2-NHL	5.46 (6.5)	446 (18.3)	0.17 (8.6)	0.004 (13.9)	0.010 (11.0)	0.011 (10.1)
CB2-CM	6.78 (4.8)	3342 (8.9)	0.18 (11.1)	0.0005 (15.7)	0.0021 (9.7)	0.003 (11.2)

## Notation

*σ_c_*	Compressive stress
*ε_c_*	Compressive strain
*σ_c,un_*	Unloading stress
*ε_c,un_*	Unloading strain
*ε_c,pl_*	Plastic strain
*ε_cu_*	Ultimate compressive strain
*σ_c,re_*	Reloading stress
*σ_c1_*	Stress at unloading level 1 in Crisafulli [26]
*σ_c2_*	Stress at unloading level 2 in Crisafulli [26]
*β_a_*	Constant in Crisafulli [26]
*ε_c,re_*	Reloading strain
*ε_cp_*	Peak strain
α, β	Constants in Subramaniam and Sinha [25]
*σ_cp_*	Peak stress
*A_1_, A_2_*	Parameters in Crisafulli [26]
*σ_c1_*	Stress at level 1
*σ_c2_*	Stress at level 2
*B_1_, B_2_, B_3_*	Parameters in Crisafulli [26]
*E_0_*	Initial elastic modulus
*E_s_*	Secant modulus
*A, D, k*	Constants in Mendola and Papia [35]
*E_s,un_*	Unloading secant modulus
*D_1_, D_2_*	Constants in Sima et al. [27]
*E_s,re_*	Reloading secant modulus
*δ_c_^−^*	Compression damage parameter in Sima et al. [27]
*δ_cu_^−^*	Unloading damage parameter in Sima et al. [27]
*n*	Ratio of initial to secant modulus
